# Strongly anisotropic in-plane thermal transport in single-layer black phosphorene

**DOI:** 10.1038/srep08501

**Published:** 2015-02-17

**Authors:** Ankit Jain, Alan J. H. McGaughey

**Affiliations:** 1 Department of Mechanical Engineering, Carnegie Mellon University, Pittsburgh, PA 15213

## Abstract

Using first principles calculations, we predict the thermal conductivity of the two-dimensional materials black phosphorene and blue phosphorene. Black phosphorene has an unprecedented thermal conductivity anisotropy ratio of three, with predicted values of 110 W/m-K and 36 W/m-K along its armchair and zigzag directions at a temperature of 300 K. For blue phosphorene, which is isotropic with a zigzag structure, the predicted value is 78 W/m-K. The two allotropes show strikingly different thermal conductivity accumulation, with phonons of mean free paths between 10 nm and 1 *μm* dominating in black phosphorene, while a much narrower band of mean free paths (50–200 nm) dominate in blue phosphorene. Black phosphorene shows intriguing potential for strain-tuning of its thermal conductivity tensor.

Two-dimensional (2D) materials (e.g., graphene, MoS_2_, silicene) are a focus of intense research because of their rich physics and potential for integration into next-generation electronic and energy conversion devices^1^,^2^,^3^,^4^. As opposed to their bulk counterparts, the optical, electronic, mechanical, and thermal properties of 2D materials can be easily tailored through the application of external strain, by introducing defects, or by stacking multiple layers of the same or different 2D materials. For example, the thermal conductivity of freely-suspended single-layer graphene is reduced from 3000–5000 W/m-K to 600 W/m-K by depositing it on amorphous SiO_2_^5^.

Recently, a new and promising 2D semiconductor, black phosphorene, was fabricated by exfoliating a few layers from bulk black phosphorus^6^,^7^. Similar to graphene, black phosphorene has a honeycomb-like structure, but it is non-planar [Fig. 1(a)]. Single-layer black phosphorene is a direct-gap semiconductor with a predicted band gap of 2 eV^8^. The band gap decreases with an increasing number of layers and is 0.3 eV for the bulk phase. Performance characteristics similar to or better than other 2D materials have been achieved for a black phosphorene-based transistor^7^. What distinguishes black phosphorene from other 2D materials is its anisotropic structure, which leads to direction-dependent optical and electronic properties that vary by as much as 50%^8^,^9^.

Our objective is to study the lattice thermal conductivity of single-layer black phosphorene, for which there is no existing experimental or theoretical data available. Ong et al. investigated the effect of strain on the ballistic thermal conductance of black phosphorene using non-equilibrium Green's functions based on harmonic lattice dynamics calculations^10^. Our aim is to predict the thermal conductivity of black phosphorene by including anharmonic phonon-phonon scattering. Thermal transport characterization is important for application in most devices, where large electrical currents can lead to Joule heating, non-radiative recombination, and potentially high operating temperatures. We find that the in-plane thermal transport in black phosphorene is strongly anisotropic, with thermal conductivity varying by a factor of three over the two orthogonal directions. While strong anisotropy in thermal conductivity is observed for van der Waals layered materials when comparing the in-plane and cross-plane directions (e.g., two orders of magnitude in graphite^11^), no other covalently-bonded 2D or 3D materials show the in-plane anisotropy we predict for black phosphorene.

We also investigate the thermal transport in blue phosphorene, another single-layered allotrope of phosphorus [Fig. 1(b)], which was recently predicted to be nearly as stable as black phosphorene^12^. First principles calculations predict a band gap in blue phosphorene in excess of 2 eV^12^, but unlike black phosphorene, blue phosphorene is isotropic.

## Methods

Thermal transport in semiconductors like black phosphorene and blue phosphorene is dominated by atomic vibrations whose energy is quantized as phonons. The phonon contribution to thermal conductivity in the *l* direction can be calculated using a solution to the Boltzmann transport equation (BTE) that uses the Fourier law, giving^13^,^14^
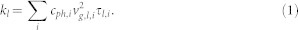
The summation in Eq. (1) is over all the phonon modes in the first Brillouin zone. The mode index, 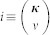, enumerates phonon wave vector, ***κ***, and polarization, *ν*. On the right-hand side of Eq. (1), *c_ph_*_,*i*_ is the volumetric specific heat, *v_g_*_,*l*,*i*_ is the *l*-component of the phonon group velocity vector **v***_g_*_,*i*_, and *τ_l_*_,*i*_ is the phonon lifetime. The phonon mean free path is |**v***_g_*_,*i*_|*τ_l_*_,*i*_. The specific heat is calculated using Bose-Einstein statistics. The group velocity vector is related to the mode frequency, 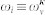, as 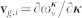. The phonon frequencies are obtained by diagonalizing the dynamical matrix and the phonon lifetimes are obtained using an iterative (full) solution of the linearized BTE for phonons^15^. The phonon-phonon scattering rates are obtained by considering three-phonon interactions in the Fermi golden rule. At a temperature of 300 K, the electronic contribution to the thermal conductivity of black phosphorene is predicted to be less than 3 W/m-K for a typical carrier concentration of 10^12^ cm^−2^ 16.

In our thermal conductivity calculations, the only required inputs are harmonic and anharmonic force constants, which are obtained from density functional theory (DFT) and density functional perturbation theory (DFPT) calculations. We use a scalar relativistic pseudopotential generated using the projector augmented-wave method as implemented in the plane wave-based quantum-chemistry package Quantum Espresso^17^. The plane wave energy cutoff is 50 Ry. To remove inter-layer interactions due to the periodicity of the computational cell, we use a vacuum of 30 Å for black phosphorene and 17 Å for blue phosphorene. For black phosphorene, the harmonic force constants are calculated on phonon and electronic wave-vector grids of 14 × 12 × 1. The cubic force constants are obtained by finite differencing of Hellmann-Feynman forces on a 144-atom supercell with a Gamma-point electronic wave-vector grid. For blue phosphorene, the electronic and phonon wave-vector grids for the harmonic force constants are 10 × 10 × 1 and the Hellmann-Feynman forces are obtained using a 128-atom supercell with a Gamma-point electronic wave-vector grid. For the thermal conductivity calculation, the phonon wave vector grid is 50 × 50 × 1 for both allotropes. Translational invariance (i.e., the acoustic sum rule) for the cubic force constants is enforced using the Lagrangian approach presented by Li et al.^18^. We note that the thermal conductivities are converged within 20% (10%) for black (blue) phosphorene for the above choice of parameters. Further details regarding these choices are provided in the supplementary information (SI).

## Results

### Phonon Dispersion

The phonon dispersion in the high-symmetry directions of the first Brillouin zone for black phosphorene and blue phosphorene are plotted in Figs. 2(a) and 2(b) and closely match those reported by Zhu et al.^12^. Black(blue) phosphorene has a four(two)-atom unit cell, resulting in twelve(six) dispersion branches. The maximum phonon frequency is similar in both allotropes (14.0 THz in black and 16.3 THz in blue), but the phonon band gap in blue phosphorene (4.9 THz) is almost double that in black phosphorene (2.5 THz). The longitudinal acoustic phonon group velocity close to the Γ point (i.e., the sound speed), is 7,733 m/s in the Γ − Y (armchair) direction and 4,168 m/s in the Γ − X (zigzag) direction for black phosphorene, an indication of anisotropic phonon transport. For blue phosphorene, the sound velocity is 8,287 m/s in both the Γ − M and Γ − K directions.

### Thermal Conductivity

**Figure 1:**
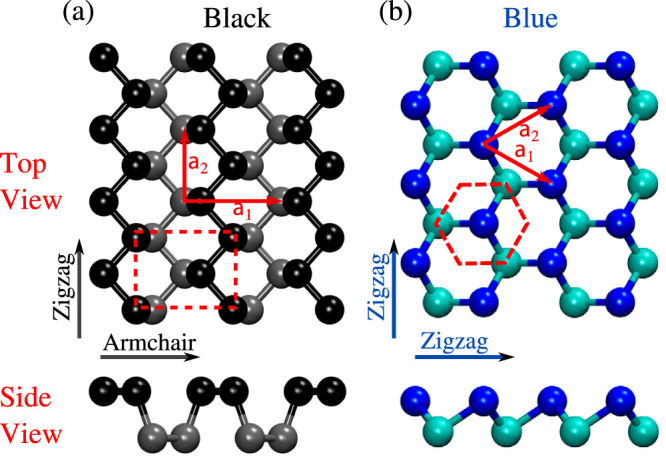
Crystal structure of (a) black phosphorene and (b) blue phosphorene. Atoms in different planes are denoted by different colors and the unit cell and the in-plane lattice vectors are shown in the top views. Black phosphorene has a four-atom unit cell with an armchair structure along **a**_1_ and a zigzag structure along **a**_2_. Blue phosphorene has a two-atom unit cell with the zigzag structure along **a**_1_ and **a**_2_. The side views show the armchair and zigzag structures. The lattice constants are |**a**_1_| = 4.43 Å and |**a**_2_| = 3.28 Å for black phosphorene, and |**a**_1_| = |**a**_2_| = 3.15 Å for blue phosphorene.

The thermal conductivities of black phosphorene and blue phosphorene for temperatures between 200 and 500 K are plotted in Fig. 3(a). For black phosphorene, thermal transport is anisotropic and we plot the thermal conductivity in both the armchair and zigzag directions [see Fig. 1(a)]. Predicting the thermal conductivity of a 2D material requires specification of the layer thickness. We choose the bulk layer separation, which is 5.25 Å for black phosphorus^19^ and 5.63 Å for blue phosphorus^12^. As the thermal conductivity scales linearly with the layer thickness, the values reported here can be easily modified for other choices.

**Figure 2:**
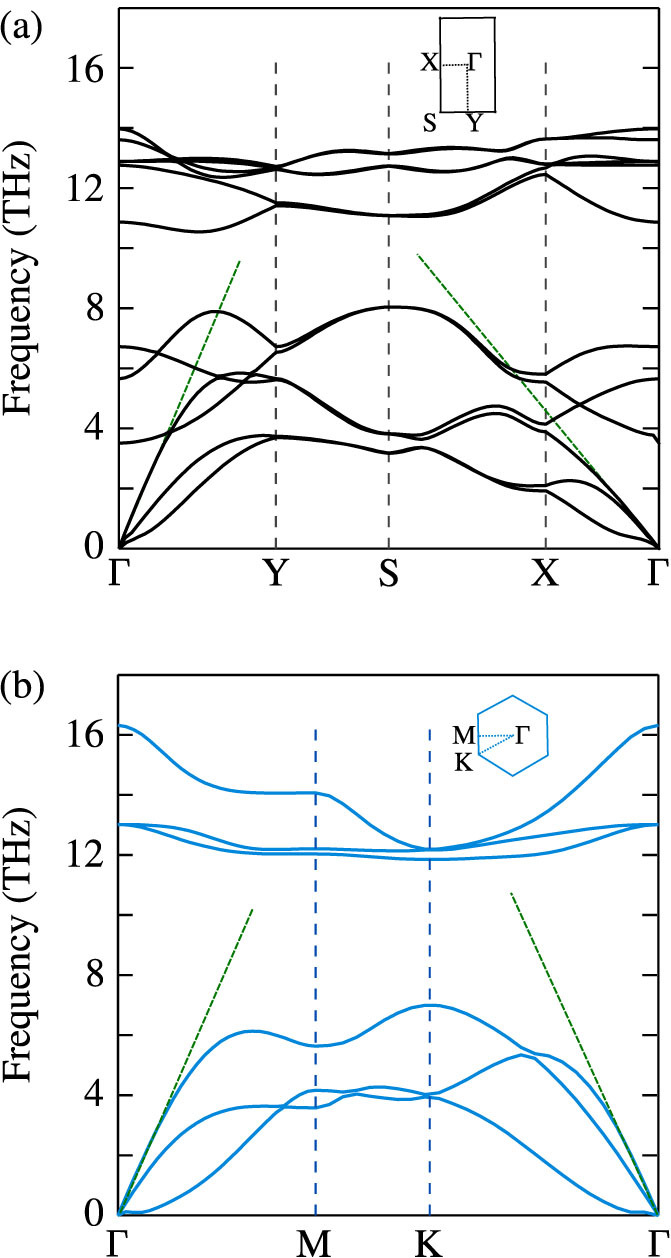
Phonon dispersion in the high symmetry directions for (a) black phosphorene and (b) blue phosphorene. The slope of the longitudinal acoustic phonon branches at the Gamma point (shown as dashed straight lines), which represents the sound velocity, is anisotropic for black phosphorene and isotropic for blue phosphorene.

Thermal conductivity decreases with increasing temperature, as expected for a phonon-dominated crystalline material. At a temperature of 300 K, the predicted thermal conductivities are 110 W/m-K (zigzag) and 36 W/m-K (armchair) for black phosphorene and 78 W/m-K for blue phosphorene. For black phosphorene, the thermal conductivity in the zigzag direction is three times higher than that in the armchair direction. This anisotropy could be useful in the design of heat channeling in micro- and nano-devices. We attribute this anisotropy in thermal conductivity to the anisotropic phonon dispersion, which leads to direction-dependent group velocities [Fig. 2(a)]. Based simply on the zone-center longitudinal acoustic group velocities, Eqn. 1 predicts a thermal conductivity anisotropy of 3.5, which is comparable to that from the full calculation. For blue phosphorene, the thermal conductivity is isotropic and is up to 1.8 times lower than the zigzag direction thermal conductivity of black phosphorene.

Our thermal conductivities are obtained using an iterative solution of the linearized BTE. For single-layer graphene, Lindsay et al. showed that the commonly used relaxation time approximation (RTA) of the BTE under-predicts the thermal conductivity by more than a factor of five at a temperature of 300 K^22^. We find that the RTA under-predicts the thermal conductivity by up to a factor of 1.3 for black phosphorene and 2.0 for blue phosphorene at a temperature of 300 K (see SI).

The thermal conductivity of black phosphorene has recently also been predicted by other researchers. Zhu et al. used the RTA to predict values of 84 W/m-K (24 W/m-K) in the zigzag (armchair) direction^20^. These values closely match our RTA predictions of 81 W/m-K (30 W/m-K) (see SI). Qin et al. predict black phosphorene thermal conductivities a factor of three lower than our values^21^. This difference may be due to (i) their use of the RTA, (ii) their choice of the cubic force constant cutoff, and/or (iii) the implementation of a translational invariance constraint on the third-order force constants. Our predictions of the effects of these factors on thermal conductivity are presented in the SI.

**Figure 3:**
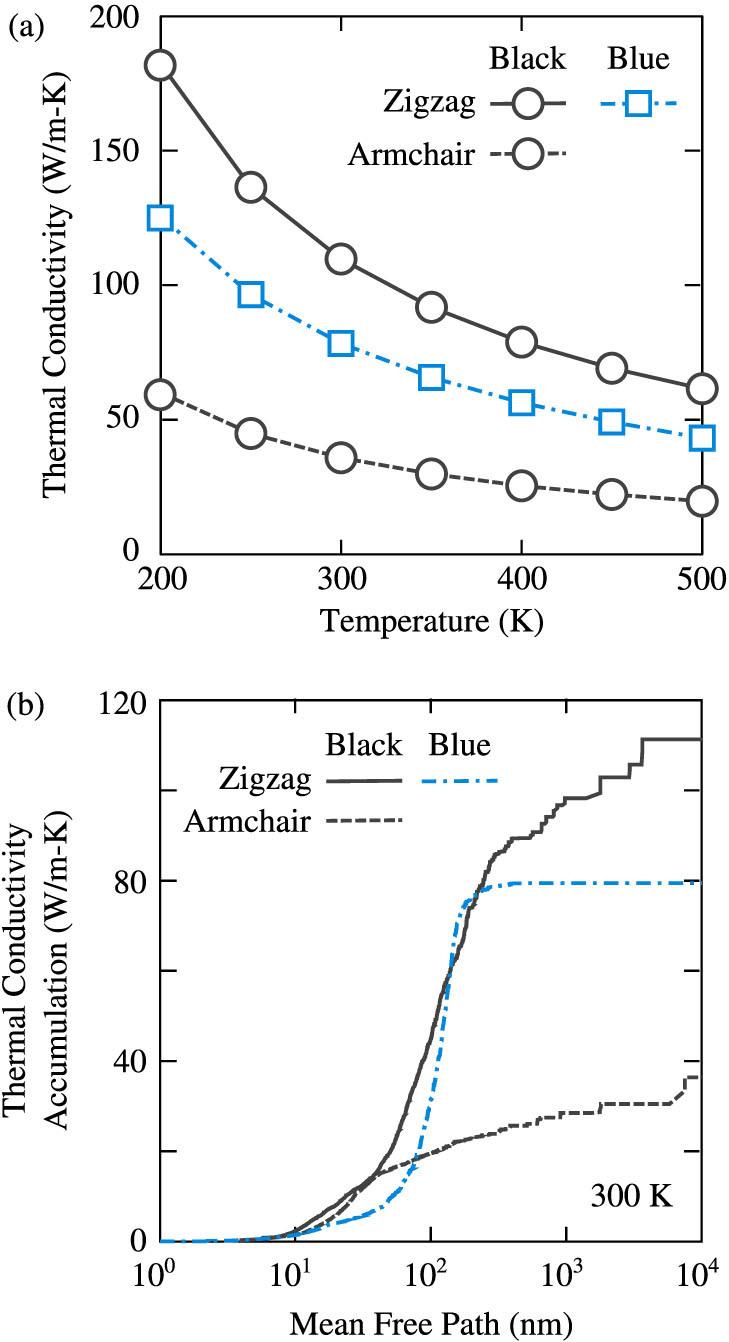
(a) Temperature-dependent thermal conductivity of black phosphorene and blue phosphorene. The thermal conductivities are obtained using an iterative solution of the linearized BTE. The predictions (symbols) are connected using lines to guide the eye. (b) Thermal conductivity accumulation functions for black phosphorene and blue phosphorene at a temperature of 300 K.

In Fig. 3(b), we plot the thermal conductivity accumulation functions for black phosphorene and blue phosphorene at a temperature of 300 K. The thermal conductivity accumulation function describes the contribution of different mean free path phonons towards the total thermal conductivity of a material^23^. Phonons with mean free paths spanning over two orders of magnitude (10 nm to 1 *μ*m) contribute towards the thermal conductivity in black phosphorene. For blue phosphorene, however, the accumulation function closely resembles a step function, with the major contribution coming from phonons with mean free paths between 50 and 200 nm. This steep thermal conductivity accumulation in blue phosphorene is similar to that in silicene (which also has buckled hexagonal structure), where phonons with mean free path between 5 and 20 nm contribute more than 80% to the thermal conductivity^24^. The thermal conductivity accumulation functions indicate that, unlike in graphene, where thermal conductivity is predicted to increase with sample sizes even greater than 10 *μ*m, changing the sample size beyond 10 *μ*m will have a minimal effect on the thermal conductivity of either phosphorene allotrope at a temperature 300 K.

As mentioned above, the structure of blue phosphorene is similar to that of silicene. Blue phosphorene, however, has a thermal conductivity of 78 W/m-K at a temperature of 300 K, which is more than eight times higher than that predicted for silicene (9.4 W/m-K)^24^. This higher thermal conductivity of blue phosphorene is the result of a larger sound velocity and the larger frequency gap in its phonon dispersion [Fig. 2(b)], which reduces the number of three-phonon scattering processes that can satisfy the energy conservation selection rule^25^,^26^,^27^,^28^. The zigzag direction thermal conductivities of black and blue phosphorene are comparable in magnitude to that predicted for a 10 *μ*m MoS_2_ sample prediction at a temperature of 300 K (108 W/m-K)^29^.

At a temperature of 300 K, Lindsay et al.^30^ predicted the thermal conductivity of graphene to be 3,600 W/m-K, which is more than 30 times higher than the thermal conductivity of either black phosphorene or blue phosphorene. We believe that the lower thermal conductivity of the phosphorene allotropes is due to their: (i) smaller sound velocities (4,000–8,000 m/s compared to 21,300 m/s in graphene^31^), (ii) lower Debye temperatures [500 K (see SI) compared to 2,300 K for graphene^32^], resulting in higher phonon-phonon scattering rates as more phonon modes are active at a given temperature, and (iii) non-planar structure, which breaks the out-of-plane symmetry. This out-of-plane symmetry exists in graphene and restricts the participation of odd numbers of ZA phonons in phonon-phonon scattering events^22^. As a result, the predicted contribution of ZA phonons to thermal conductivity in the phosphorene allotropes varies from 12–44% as compared to 76% in graphene at a temperature of 300 K^30^, as presented in Table 1. We note that the thermal conductivity contributions of the different acoustic phonon branches in black phosphorene and blue phosphorene are similar to those predicted for MoS_2_^29^, which also has a non-planar structure. More information is provided in the SI.

The thermal conductivity of freely-suspended single-layer graphene at a temperature of 300 K is reduced from 3000–5000 W/m-K to 600 W/m-K when it is deposited on amorphous SiO_2_^5^. This more than a factor of five reduction in the thermal conductivity is due to the increased scattering of ZA phonons in supported graphene. The results presented in this work are for suspended phosphorene. As can be seen in Table 1, the contribution of ZA phonons is 31% (12%) in the zigzag (armchair) direction in black phosphorene and 44% in blue phosphorene. We expect a comparable decrease in the thermal conductivity of supported phosphorene samples.

### Strain Tuning of Thermal Conductivity

**Figure 4:**
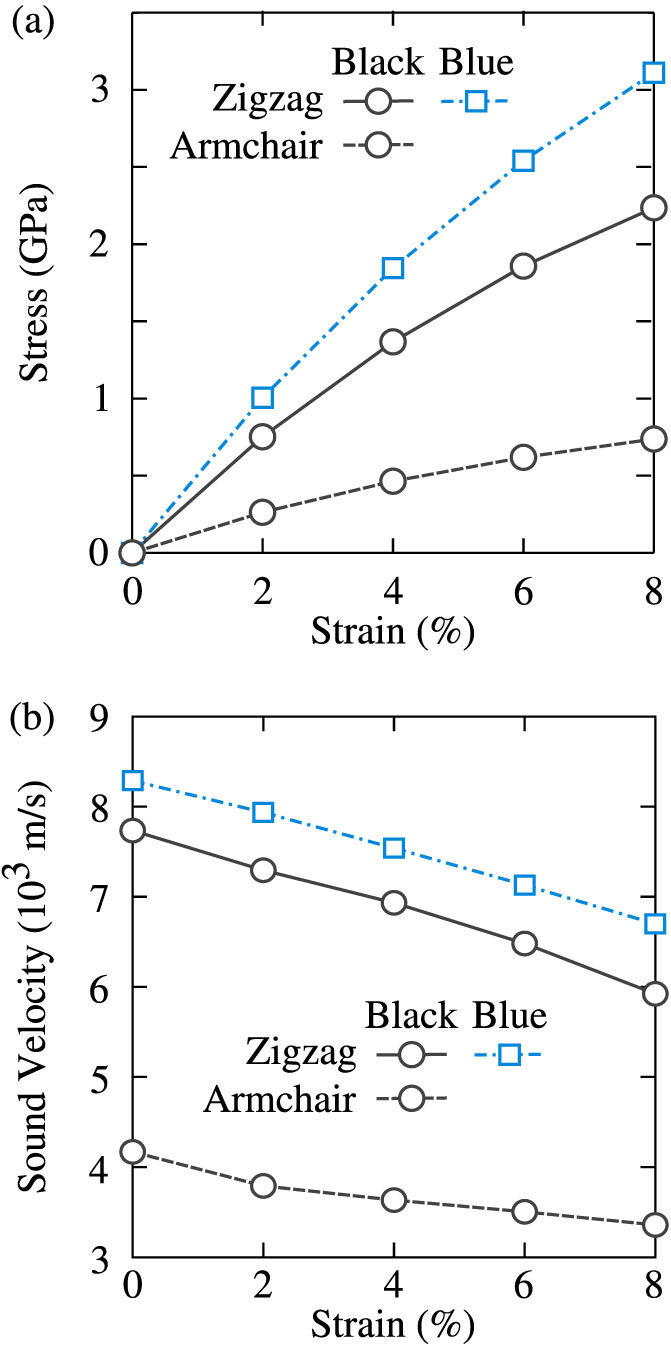
(a) Stress and (b) sound velocity in black phosphorene and blue phosphorene under bi-axial strain. The predicted values (symbols) are connected using lines to guide the eye.

We now consider the possibility of strain-tuning the thermal conductivity of black phosphorene and blue phosphorene. In Fig. 4(a), we plot the stresses in both allotropes when they are subjected to a bi-axial tensile strain. For black phosphorene, the stress is anisotropic and is three times lower in the armchair direction compared to the zigzag direction. For blue phosphorene, which has a zigzag structure, the stress is isotropic and is 1.5 times larger than the stress in the zigzag direction of black phosphorene.

As can be seen from Eqn. 1, thermal conductivity scales as the square of the phonon group velocities. We plot the strain-dependence of the sound velocities in Fig. 4(b) for black phosphorene and blue phosphorene under the bi-axial strain conditions. The sound velocities are maximum at zero strain for both materials and decrease with increasing strain. Using this reduction as a guide, we estimate that the thermal conductivities of both allotropes may decrease by a factor of 1.7 at a strain of 8%. Similar calculations for uni-axial strains suggest the possibility for strain-tailoring the thermal conductivity anisotropy in black phosphorene. For example, the estimated anisotropy in the thermal conductivity of black phosphorene is a factor of 4.6 (2.2) for uni-axial strain of 6% along the armchair (zigzag) direction (see SI).

### Anisotropy

To the best of our knowledge, no other 2D material displays anisotropic in-plane thermal transport. Furthermore, the thermal conductivity anisotropy in black phosphorene is striking when compared to that found in layered, wurtzite, and orthorhombic three-dimensional crystal structures, as compiled in Table 2. We quantify anisotropy, *r*, by the ratio of the maximum and minimum direction-dependent thermal conductivities. The maximum *r* is in the layered structures graphite and hexagonal boron nitride. These layers are weakly bonded through van der Waals interactions compared to the strong covalent bonds within the layers, which results in poor interlayer heat transport. Of the remaining materials in Table 2, which are all covalently bonded, the maximum anisotropy is for our prediction for black phosphorene, which is twice as large as the next highest value.

## Summary

We predicted the thermal conductivity of black phosphorene and blue phosphorene using first-principles-driven lattice dynamics calculations and a full (iterative) solution of the BTE. We found a factor of three anisotropy in the thermal conductivity of black phosphorene, which could potentially be tuned up to 4.6 using strain. At a temperature of 300 K, the predicted thermal conductivities of both phosphorene allotropes are larger than that of silicene, similar to that of MoS_2_, and are up to two order of magnitude smaller than that of graphene.

## Author Contributions

A.J. performed the calculations. A.J. and A.J.H.M. analyzed the data. A.J. wrote the manuscript. A.J.H.M. reviewed and edited the manuscript.

## Supplementary Material

Supplementary Information Supplementary Information

## Figures and Tables

**Table 1:** Contribution of different phonon modes branches [longitudinal acoustic (LA), transverse acoustic (TA), out-of-plane acoustic (ZA), and all optical] towards the total thermal conductivity in black phosphorene, blue phosphorene, MoS_2_ (10 *μ*m sample^29^), and graphene (10 *μ*m sample^30^) at a temperature of 300 K

Material	Total thermal conductivity (W/m-K)	LA (%)	TA (%)	ZA (%)	Optical (%)
Black phosphorene (zigzag)	110	32	22	31	15
Black phosphorene (armchair)	36	28	33	12	27
Blue phosphorene	78	26	27	44	3
MoS_2_^29^	108	28	24	39	9
Graphene^30^	3600	8	15	76	1

**Table 2:** Anisotropy in thermal conductivity for selected materials with layered, wurtzite, and orthorhombic crystal structure at a temperature of 300 K. ‘exp' and ‘pred' in the first column denote experimental measurements and simulation predictions

Material	*k*_max_ (W/m-K)	*k*_min_ (W/m-K)	*r*
Graphite (exp^33^)	(1000–2000)	6	~300
h-BN (exp^33^)	(200–300)	2	~100
**Black Phosphorene**	**110**	**36**	**3.1**
SnSe (exp^34^)	0.70	0.46	1.52
GaN (pred^14^)	401	385	1.04
AlN (pred^14^)	322	303	1.06
NdFeO_3_ (pred^35^)	3.01	2.68	1.12
NdAlO_3_ (pred^35^)	6.61	5.72	1.16
