# Supplementary material for: Strongly anisotropic in-plane thermal transport in single-layer black phosphorene
Source: Sci Rep. 2015 Feb 17;5:8501. doi: 10.1038/srep08501 (PMC4330521; doi:10.1038/srep08501)
Supplement: Supplementary Information [file srep08501-s1.pdf]

Supplementary Information for "Strongly anisotropic in-plane thermal transport in single-layer black phosphorene"

Ankit Jain[1] & Alan. J. H. McGaughey[1]*

[1]*Department of Mechanical Engineering, Carnegie Mellon University, Pittsburgh PA 15213*

**mcgaughey@cmu.edu*

1 Thermal Conductivity Convergence

Predicting thermal conductivity from lattice dynamics calculations and the Boltzmann transport equation (BTE) requires harmonic and anharmonic force constants as input. We obtain these harmonic and anharmonic force constants from first-principles-driven density functional perturbation theory (DFPT) and density functional theory (DFT) calculations. The calculation of thermal conductivity from the DFPT and DFT force constants requires specification of the supercell size, the force interaction cutoffs, and, for 2D materials like black phosphorene and blue phosphorene. the amount of vacuum needed to remove inter-layer interactions. In this section, we discuss the convergence of the thermal conductivity of black phosphorene and blue phosphorene with these calculation parameters.

Cubic Force Constant Cutoff

In Figs. S1(a) and S1(b), we plot the thermal conductivity of black phosphorene and blue

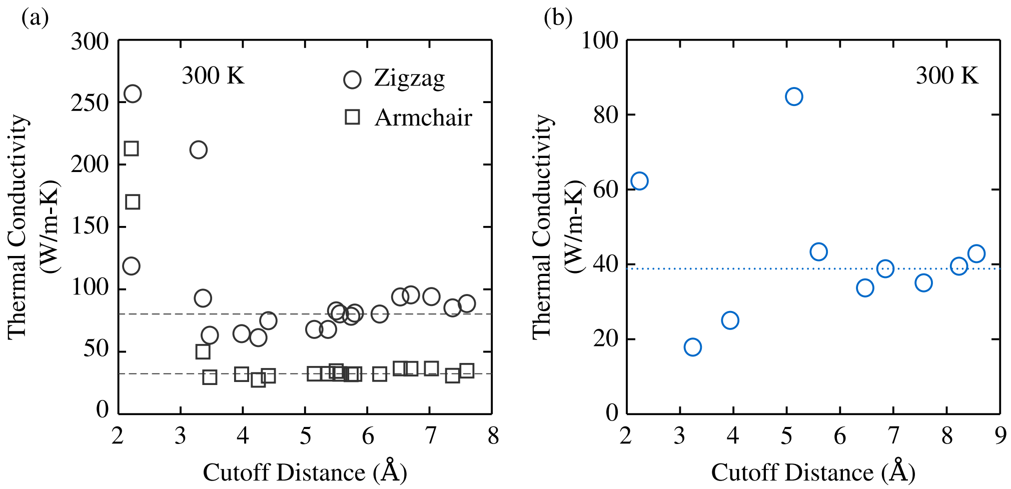

Figure S1: Thermal conductivity variation of (a) black phosphorene and (b) blue phosphorene with the cubic force constant interaction cutoff at a temperature of 300 K.

phosphorene as a function of the cubic force-constant interaction cutoff at a temperature of 300 K. The cubic force constants are obtained using 144 (128) atom supercells with 30 Å (17 Å) of vacuum for black (blue) phosphorene. Translational invariance (TI) is satisfied using the Lagrangian approach presented by Li et al.[1] The thermal conductivities in these figures are predicted using the relaxation time approximation (RTA) solution of the BTE.

As can be seen from Figs. S1(a) and S1(b), the thermal conductivity for both black phosphorene and blue phosphorene converges beyond an interaction cutoff of 5.5 Å. In all reported calculations, we use a interaction cutoff of 6.2 Å for black phosphorene and 6.5 Å for blue phosphorene. The predicted thermal conductivities changes by 12% (10%) when the interaction cutoff is increased from 6.2 Å to 7.6 Å (6.5 Å to 8.6 Å) for black (blue) phosphorene.

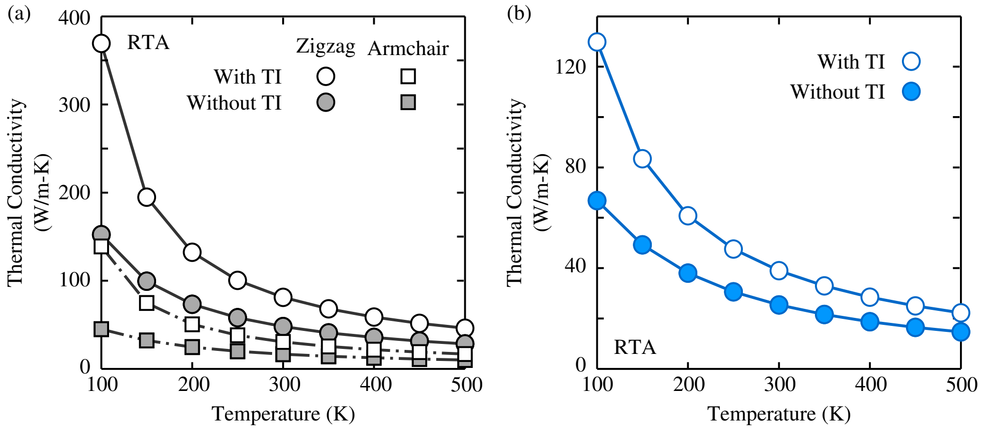

Figure S2: The effect of translational invariance on the thermal conductivity of (a) black phosphorene and (b) blue phosphorene.

DFT Supercell Size and Vacuum Width

To determine the effect of supercell size and vacuum width on the predicted thermal conductivities of black phosphorene and blue phosphorene, we compared cubic force constants calculated using two different supercell sizes and vacuum widths. For black phosphorene, we compared cubic force constants calculated on a 144 atom supercell with 30 Å of vacuum to those from a 100 atom supercell with 20 Å of vacuum. The two sets of force constants were found to be very close, with differences of less than 2% for the largest (i.e., the self) force constant. For blue phosphorene, we compared force constants calculated on 128 and 98 atom supercells with 17 Å of vacuum and found a difference of less than 1% for the largest force constant.

Translational Invariance of Cubic Force Constants

The cubic force constants obtained using finite differences of the Hellman-Feynman forces

do not satisfy crystal symmetries and translational invariance (TI) (i.e., the acoustic sum rule) because of numerical errors. These small numerical errors can result in large changes in thermal conductivity predictions, as shown by Lindsay et al.[2]. It is therefore necessary to satisfy the crystal TI constraint by modifying the cubic force constants. In this study, we satisfied this TI constraint using the Lagrangian approach presented by Li et al.[1]

The effect of TI on the thermal conductivity (calculated using the RTA) of black phosphorene and blue phosphorene is shown in Figs. S2(a) and S2(b). At a temperature of 300 K, not satisfying the cubic TI constraint results in an under-prediction of thermal conductivity by 40% (47%) in the zigzag (armchair) direction of black phosphorene and by 33% in blue photosphere. We note that the effect of not satisfying TI is more severe in phosphorene, as compared to that reported by Lindsay et al.[2] for Ge (29% at 300 K), because of fewer crystal symmetries in phosphorene.

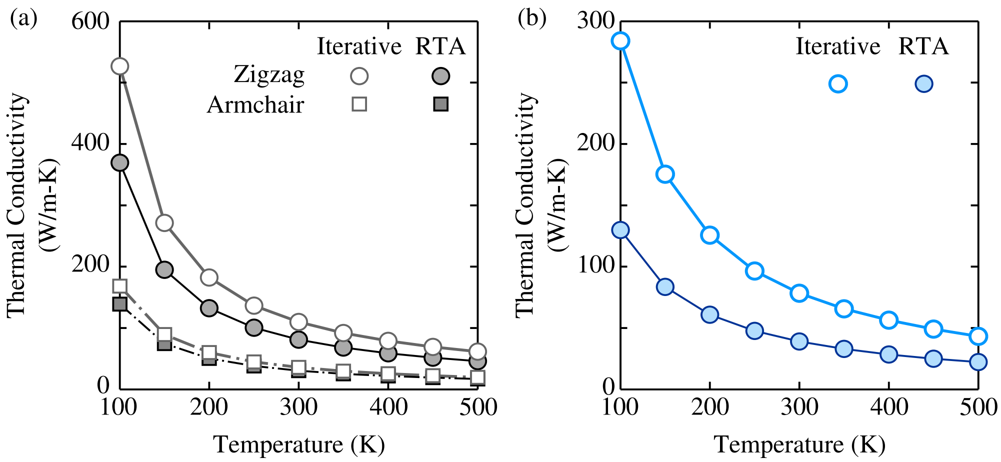

Figure S3: Thermal conductivity prediction of (a) black phosphorene and (b) blue phosphorene using the RTA and iterative (full) solution of the BTE.

RTA Versus Full Solution of the BTE

The thermal conductivity variation of black phosphorene and blue phosphorene using the RTA and an iterative solution of the BTE are plotted as a function of temperature in Figs. S3(a) and S3(b). The cubic force constants are obtained using the 144 atom supercell with 30 Å of vacuum for black phosphorene and the 128 atom supercell with 17 Å of vacuum for blue phosphorene. The TI for cubic force constants is satisfied as discussed in the previous section.

As can be seen from Figs. S3(a) and S3(b), the RTA solution of the BTE under-predicts the thermal conductivity for both black phosphorene and blue phosphorene. At a temperature of 300 K, the under-predictions are by a factor of 1.4 and 1.2 in the zigzag and armchair directions for black phosphorene and a factor of 2.0 for blue phosphorene. As explained by Lindsay et al.[3] for graphene, these under-predictions in thermal conductivity are due to Normal phonon processes

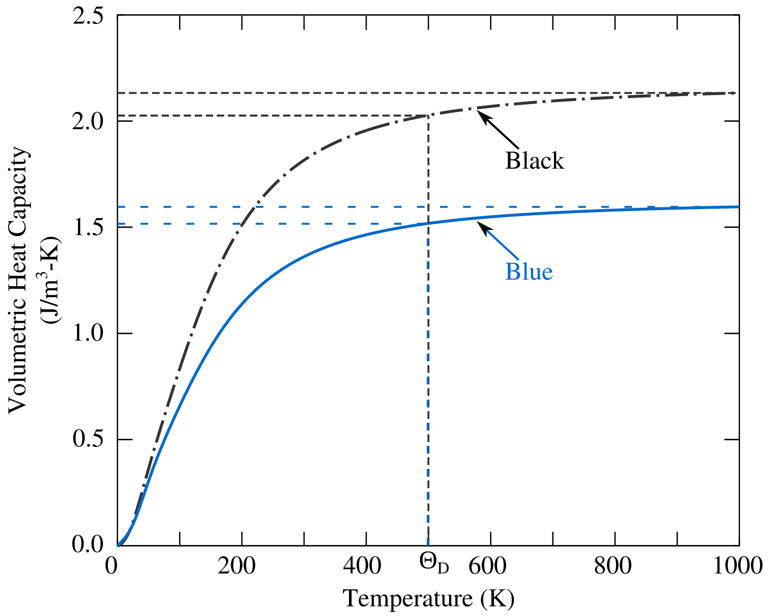

Figure S4: Volumetric heat capacity variation of black phosphorene and blue phosphorene with temperature. The dashed vertical line corresponds to the temperature at which the heat capacity is 95% of its maximum value (i.e., the Debye temperature).

being treated as resistive in the RTA solution of the BTE.

2 Debye Temperature

In Fig. S4, we plot the volumetric heat capacity variation of black phosphorene and blue phosphorene with temperature. In our calculations, we considered only the phonon (lattice) contribution towards the heat capacity. At a temperature of 300 K, the heat capacity of blue phosphorene is 0.75 times the heat capacity of black phosphorene. We estimate the Debye temperature as the temperature at which the heat capacity is 95% of its maximum value. For both phosphorene allotropes, the

Debye temperature is 500 K.

3 Contribution of Acoustic Phonon Branches to Thermal Conductivity

We plot the contribution of the different acoustic phonon beaches towards the total thermal conductivity as a function of temperature in Figs. S5(a) [black phosphorene (zigzag)], S5(b) [black phosphorene (armchair)], and S5(c) (blue phosphorene). For black phosphorene, the maximum contribution is from longitudinal acoustic (LA) phonon modes in the zigzag direction and transverse acoustic (TA) phonon modes in the armchair direction. The contribution of out-of-plane (ZA) phonon modes in black phosphorene remains constant with temperature at around 31% (12%) for the zigzag (armchair) direction of heat flow. For blue phosphorene, the maximum contribution comes from the ZA phonon modes and is more than 42% for the range of temperatures considered.

4 Sound Velocity Variation with Uni-axial Strain

In Figs. S6(a) and S6(b), we plot the stress generated and sound velocity in the zigzag and armchair direction of black phosphorene when it is subjected to a uni-axial strain along the zigzag direction. The sound velocity decreases by 4.5% along the zigzag direction and increases by 20% in the armchair direction when strain is changed from 0 to 6% along the zigzag direction. These changes in the sound velocity suggest (according to Eqn. 1 in the main text) a reduction in the thermal transport anisotropy to 2.2 for 6% strain along the zigzag direction.

For uni-axial strain along the armchair direction [Figs. S6(c) and S6(d)] in black phospho-

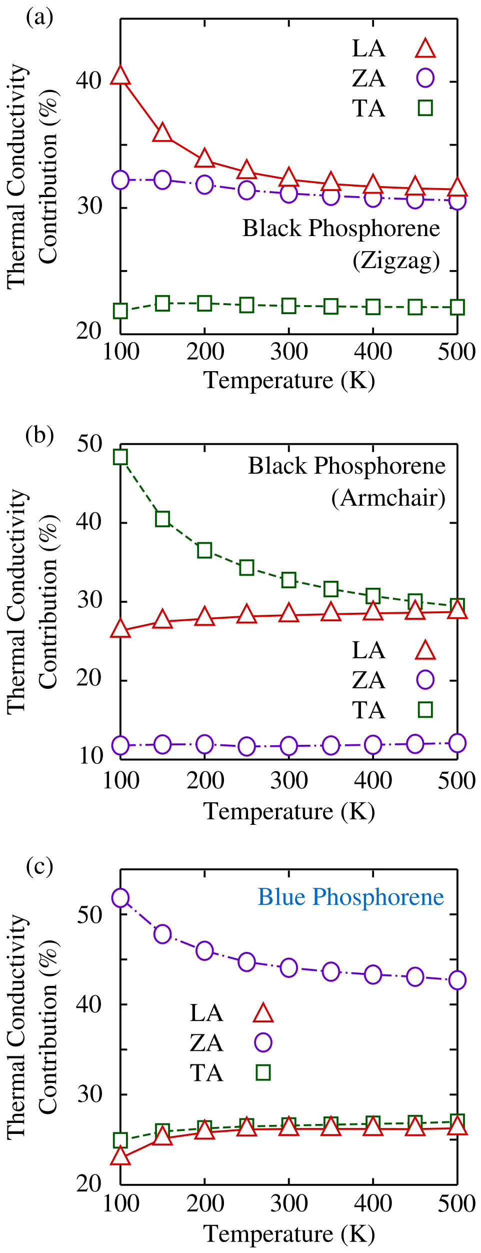

Figure S5: Contribution of acoustic phonon branches [longitudinal acoustic (LA), transverse acoustic (TA), and out-of-plane acoustic (ZA)] towards the total thermal conductivity of (a) black phosphorene in the zigzag direction, (b) black phosphorene in the armchair direction, and (c) blue phosphorene.

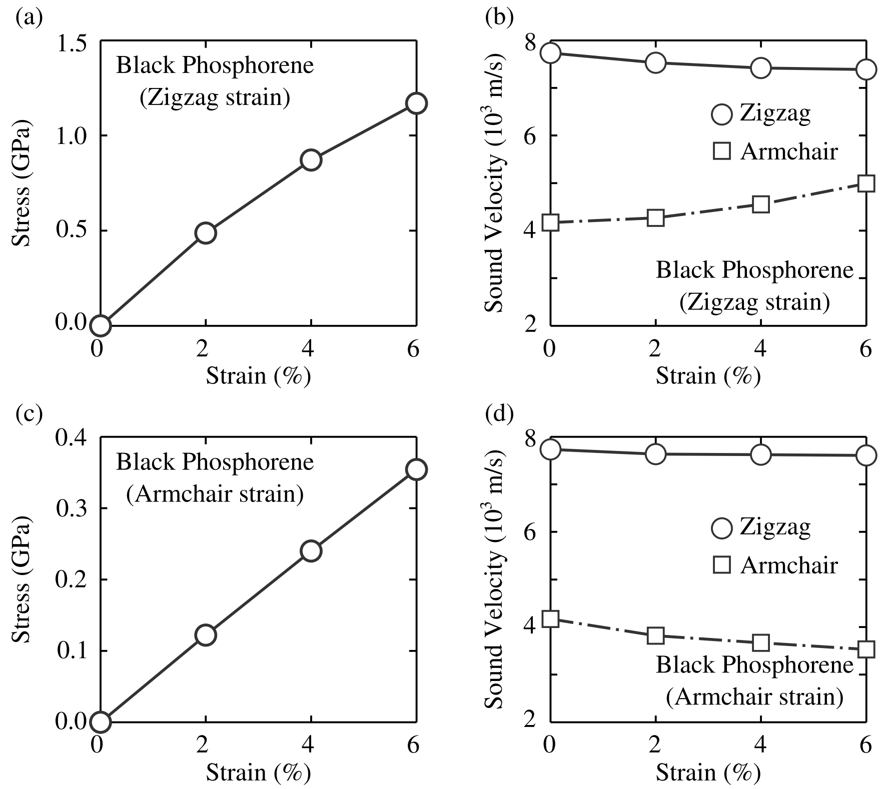

Figure S6: Variation of stress and sound velocity in black phosphorene with uni-axial strain along the zigzag [(a) and (b)] and the armchair [(c) and (d)] directions.

rene, the sound velocity decreases by only 2% in the zigzag direction and 15% in the armchair direction when strain is changed from 0 to 6%. The thermal conductivity anisotropy, therefore, is estimated to increase to 4.6 under 6% uni-axial strain in black phosphorene along the armchair direction.

1. Li, W., Lindsay, L., Broido, D. A., Stewart, D. A. & Mingo, N. Thermal conductivity of bulk and nanowire $mg_2si_xsn_1$-x alloys from first principles. *Phys Rev B* **86**, 174307 (2012). URL http://link.aps.org/doi/10.1103/PhysRevB.86.174307.

2. Lindsay, L., Broido, D. A. & Reinecke, T. L. *Ab initio* thermal transport in compound semiconductors. *Phys Rev B* **87**, 165201 (2013). URL http://link.aps.org/doi/10.1103/PhysRevB.87.165201.

3. Lindsay, L., Broido, D. A. & Mingo, N. Flexural phonons and thermal transport in graphene. *Phys Rev B* **82**, 115427 (2010). URL http://link.aps.org/doi/10.1103/PhysRevB.82.115427.
